# A Bayesian approach to study the risk variables for tuberculosis occurrence in domestic and wild ungulates in South Central Spain

**DOI:** 10.1186/1746-6148-8-148

**Published:** 2012-08-30

**Authors:** Víctor Rodríguez-Prieto, Beatriz Martínez-López, José Ángel Barasona, Pelayo Acevedo, Beatriz Romero, Sabrina Rodriguez-Campos, Christian Gortázar, José Manuel Sánchez-Vizcaíno, Joaquín Vicente

**Affiliations:** 1 VISAVET, Veterinary School, Complutense University of Madrid, Puerta de Hierro s/n, Madrid 28040, Spain; 2 IREC Instituto de Investigación en Recursos Cinegéticos (CSIC-UCLM-JCCM), Ronda de Toledo s/n, Ciudad Real, 13071, Spain; 3 Biogeography, Diversity and Conservation Research Team, University of Malaga, Málaga, 29071, Spain; 4 Centre de Recerca en Sanitat Animal (CReSA), UAB-IRTA, Campus de la Universitat Autònoma de Barcelona, Bellaterra (Cerdanyola del Vallés), 08193, Spain

## Abstract

**Background:**

Bovine tuberculosis (bTB) is a chronic infectious disease mainly caused by *Mycobacterium bovis*. Although eradication is a priority for the European authorities, bTB remains active or even increasing in many countries, causing significant economic losses. The integral consideration of epidemiological factors is crucial to more cost-effectively allocate control measures. The aim of this study was to identify the nature and extent of the association between TB distribution and a list of potential risk factors regarding cattle, wild ungulates and environmental aspects in Ciudad Real, a Spanish province with one of the highest TB herd prevalences.

**Results:**

We used a Bayesian mixed effects multivariable logistic regression model to predict TB occurrence in either domestic or wild mammals per municipality in 2007 by using information from the previous year. The municipal TB distribution and endemicity was clustered in the western part of the region and clearly overlapped with the explanatory variables identified in the final model: (1) incident cattle farms, (2) number of years of veterinary inspection of big game hunting events, (3) prevalence in wild boar, (4) number of sampled cattle, (5) persistent bTB-infected cattle farms, (6) prevalence in red deer, (7) proportion of beef farms, and (8) farms devoted to bullfighting cattle.

**Conclusions:**

The combination of these eight variables in the final model highlights the importance of the persistence of the infection in the hosts, surveillance efforts and some cattle management choices in the circulation of *M. bovis* in the region. The spatial distribution of these variables, together with particular Mediterranean features that favour the wildlife-livestock interface may explain the *M. bovis* persistence in this region. Sanitary authorities should allocate efforts towards specific areas and epidemiological situations where the wildlife-livestock interface seems to critically hamper the definitive bTB eradication success.

## Background

Bovine tuberculosis (bTB) is a chronic infectious disease that causes significant economic losses worldwide. *Mycobacterium bovis*, the main causative agent of the disease, is transmitted between a variety of domestic and wild animals, and humans [1]. *M. bovis* is primarily transmitted by aerosols and may persist for long periods in the environment, especially in moist and shady zones, such as the water points or the woodlands [2]. Due to its widespread distribution and the sanitary and economic impact, eradication of bTB is considered a priority in the European Union [3]. A great amount of personal and financial resources are invested every year in order to control the disease. Control and preventive sanitary programs for bTB eradication have been conducted for decades and traditionally focused on cattle, mainly consisting of culling reactor animals to the intradermal tuberculin test (IDTT). Despite these efforts, the disease remains prevalent or even increasing in many regions of the world, most likely associated to wild mammal reservoirs [4,5].

Several studies have highlighted the role of certain wildlife species as major reservoirs, depending on the ecosystem and the region of the world [6,7]. Among the wild mammals in Spain, the Eurasian wild boar (*Sus scrofa*) and the red deer (*Cervus elaphus*) constitute the most important reservoirs, especially in Mediterranean areas, because they are able to maintain and efficiently transmit the agent in the absence of other domestic or wild hosts [8-10]. The implication of wildlife species in this multi-host cycle, in addition to many environmental and ecological factors, probably hampers the success in the control of bTB and makes the complete eradication of the disease currently not feasible [11-13].

Despite the national and long-lasting coverage of the bTB eradication plans in Spain, the prevalence of bTB varies considerably between regions [14]. In 2009, while disease presence was negligible in the islands and low in the North-East part of the country (prevalence between 0.21 and 0.91%), the herd prevalence in some regions of central, southern and western Spain was considerably higher, reaching values up to 10.27% in Castile-La Mancha [14-16]. There are several epidemiological circumstances that might be favouring this situation. First, cattle herds are farmed especially for beef (and to a lesser extent for bullfighting), comprising breeds of animals difficult to deal with due to their strong character. This kind of cattle is managed in extensive Mediterranean conditions in areas with difficult access, unfavourable topography and lack of resources, which makes it quite difficult to properly test all the animals of the herd. This also induces common grazing and a shared use of scarce water points (such as water holes), and thus, the contact of cattle with other domestic livestock such as sheep, goats, pigs [15] and wildlife [17]. Secondly, some areas, especially in the South Central regions, have experienced a great development in the recreational hunting industry, which leads to overabundance of wild ungulates in which sanitary programs cannot be implemented, leading to high prevalence of diseases [9,18]. All these aspects promote the existence of a wildlife-domestic livestock interface in Mediterranean habitats that could maintain a multihost environmental reservoir for *M. bovis*[11,13]. However, there is no research on the association between these factors and the status of bTB at either fine or large spatial scales.

In fact, regional variations in bTB eradication strategies through Spain are mainly based on the cattle animal/herd prevalence or incidence rates, data obtained from the National Programmes for bTB eradication [15]. The first contribution of the risk concept to the sanitary strategies appears in the literature only very recently [14], although there is a lack of inclusion and evaluation of wildlife-related risk factors. Moreover, the marked spatial variability in bTB risks indicates that we need detailed approaches in particular areas to evaluate different cattle management, environmental and biogeographical factors [19]. Characterization of risk factors for TB occurrence are crucial to more cost-effectively allocate financial and personal resources in order to improve the preventive, control and eradication measures in Spain and other regions with similar epidemiological conditions. Here we focus on an area from South Central Spain (Ciudad Real province) characterized by a complex epidemiological scenario for bTB, with the existence of a wildlife-domestic livestock interface under Mediterranean conditions and presence of abundant, widely distributed wild ungulate populations where TB is endemic and highly prevalent [9]. In this epidemiological context we used a Bayesian model to explore the nature and extent of the association between the distribution of *M. bovis* infection and potential risk factors in relation to cattle, wild ungulates and environmental aspects. This study is intended to guide policy makers towards risk-based and cost-effective TB eradication strategies.

## Results

**Figure 1:**
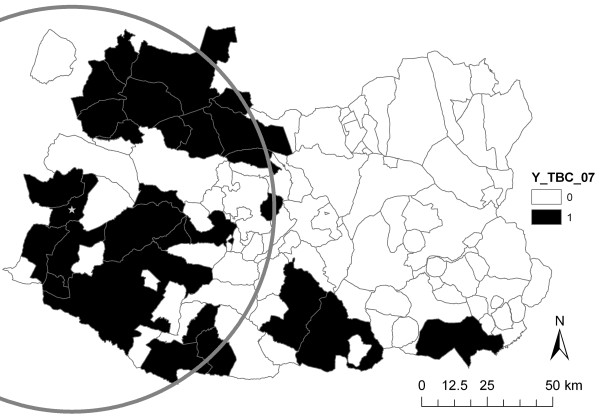
**Spatial distribution of TB occurrence in Ciudad Real in 2007.** The grey circle marks the significant cluster obtained by the Bernoulli model (star denoting its centre).

**Figure 2:**
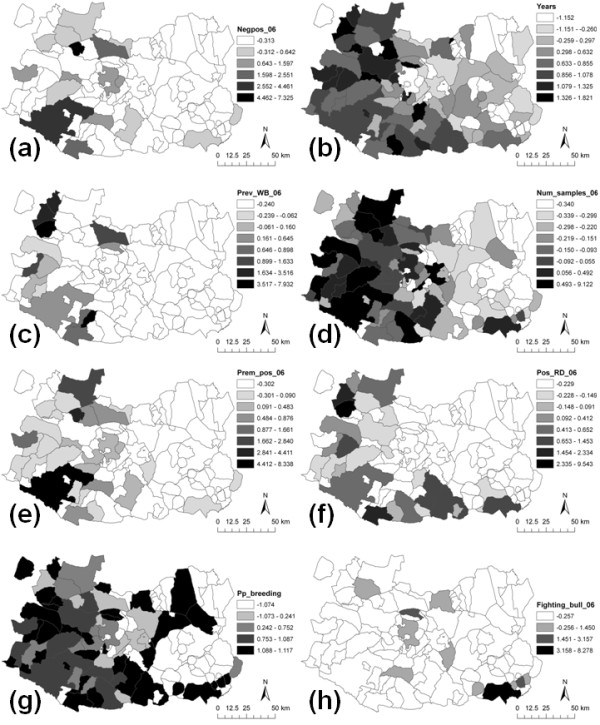
**Spatial distribution of the eight variables retained in the final model.** Namely, (**a**) the proportion of cattle farms becoming bTB-positive in 2006, (**b**) the mean of hunting seasons in which the hunting estates of the municipality have been inspected, (**c**) the apparent prevalence of TB in wild boar in 2006, (**d**) the number of sampled cattle in the cattle farms included in the sanitary plan in 2006, (**e**) the number of bTB-positive cattle farms in 2006, (**f**) the number of TB-positive red deer in 2006, (**g**) the proportion of cattle farms classified as breeding farms in 2006, and (**h**) the number of farms for bullfighting cattle in 2006.

TB occurrence in Ciudad Real municipalities was spatially clustered in 2007, as indicated by the visual inspection of the spatial distribution of disease and the spatial cluster identified (RR = 12.88) (Figure 1). The model with no predictors (i.e. only with the intercept and structured and unstructured random effects) had a DIC of 101.74. The final model that best fitted the data (DIC = 72.32; *pD* = 5.225) included 8 variables (Table 1); three of them (namely, the proportion of cattle farms becoming bTB positive in 2006, the mean number of hunting seasons in which the hunting estates of the municipality have been inspected, and the apparent prevalence of TB in wild boar in 2006) were statistically significant (95% CI) and positively (OR > 1) associated with TB occurrence (Table 1). The number of sampled animals in the cattle farms (i.e. proxy of the total census) included in the sanitary plan in 2006, the number of cattle farms with at least one bTB-positive animal in 2006, the number of TB-positive red deer in 2006, and the proportion of cattle farms classified as breeding farms in 2006 were marginally statistically significant (90% CI, OR > 1). The number of farms for bullfighting cattle appeared to improve to final model, although it was not highly significant. The spatial distribution of the variables included in the final model is shown in Figure 2.

**Table 1:** **Beta coefficients and Odds ratios for the final model**.

	**Type of variable**	**Beta coefficients**			**Odds ratios**		
		**Median**	**95% CI**	**90% CI**	**Median**	**95% CI**	**90% CI**
Intercept		−1.07	[−1.53, -0.64]	[−1.42, -0.73]	0.34	[0.22, 0.53]	[0.24, 0.48]
Proportion of cattle farms becoming bTB positive from 2005 to 2006 (%)	QS	0.62^(a)(b)^	[0.06, 1.22]	[0.18, 1.09]	1.86^(a)(b)^	[1.06, 3.39]	[1.19, 2.97]
Mean number of hunting seasons in which the hunting estates of the municipality have been inspected	QS	0.55^(a)(b)^	[0.09, 1.02]	[0.19, 0.91]	1.73^(a)(b)^	[1.09, 2.77]	[1.21, 2.49]
Apparent TB prevalence in wild boars in the municipality in the game season 2006-07	QS	0.66^(a)(b)^	[0.18, 1.28]	[0.27, 1.14]	1.94^(a)(b)^	[1.20, 3.59]	[1.32, 3.11]
Number of sampled cattle in the cattle farms included in the sanitary plan in 2006	QS	0.61^(b)^	[−0.05, 1.30]	[0.10, 1.14]	1.85^(b)^	[0.96, 3.66]	[1.10, 3.13]
Number of cattle farms with at least one bTB-positive animal in 2006	QS	0.66^(b)^	[−0.04, 1.38]	[0.11, 1.22]	1.94^(b)^	[0.96, 3.98]	[1.11, 3.40]
Number of “TB-positive” red deers in the municipality in the game season 2006-07	QS	0.53^(b)^	[−0.08, 1.22]	[0.05, 1.06]	1.69^(b)^	[0.93, 3.38]	[1.05, 2.90]
Proportion of cattle farms classified as extensive beef breeding farms in 2006 (%)	QS	0.39^(b)^	[−0.09, 0.87]	[0.01, 0.76]	1.47^(b)^	[0.92, 2.39]	[1.02, 2.14]
Number of farms devoted to bullfighting cattle in 2006	QS	0.14	[−0.24, 0.56]	[−0.16, 0.47]	1.15	[0.79, 1.76]	[0.85, 1.60]

(QS) = quantitative standardized variable; (D) = dichotomous variable (codified considering the median).

(a): Significant coefficients of the final model using the 95% CI.

(b): Significant coefficients of the final model using the 90% CI.

**Figure 3:**
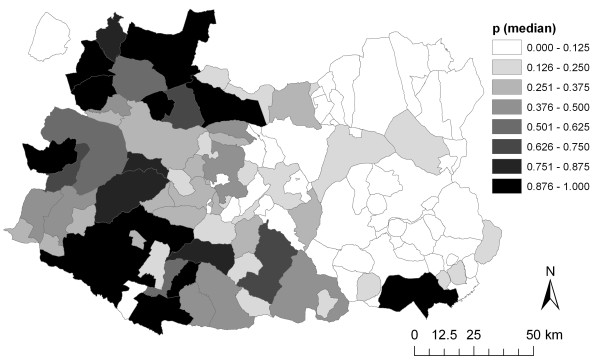
**Posterior (predicted) probability (p**_**i**_**) of TB occurrence in Ciudad Real estimated by the model.**

**Figure 4:**
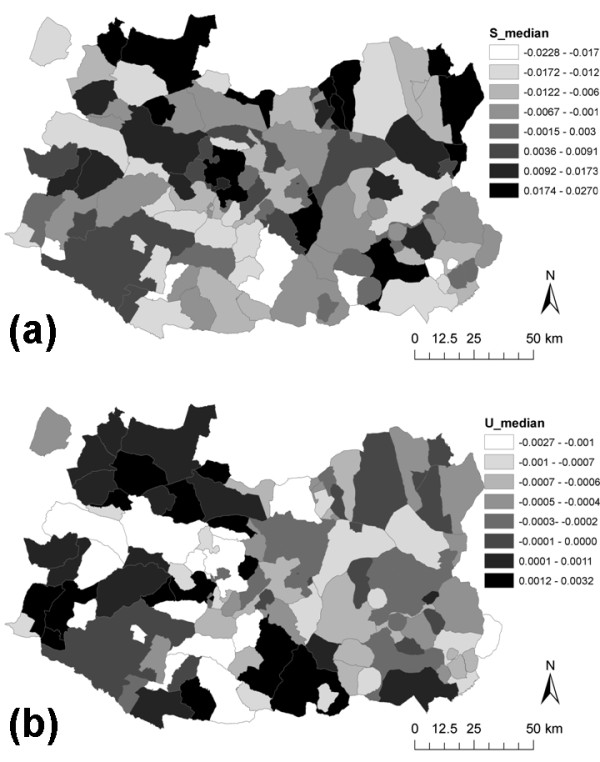
**Distribution of the spatially (a) structured random effects (S**_**i**_**) and (b) unstructured random effects (U**_**i**_**) included in the Bayesian multivariate logistic regression mixed model for TB occurrence in Ciudad Real.**

The posterior probability of TB occurrence in Ciudad Real estimated by the best fitting model resembled the spatial structure of the disease observed in the data, with areas at the highest risk concentrated in the Northwest corner, the Southwest part and several municipalities in the southern stripe of the region (Figure 3). Both S_i_ and U_i_ were very low (median value of −2.33·10^-3^ and −2.96·10^-4^, respectively), which may indicate no significant presence in the model of spatial autocorrelation and overdispersion over-and-above the risk factors identified, as well as a good fit of the model (Figure 4). In addition, convergence of the model was obtained after the first 200 iterations and no problems with autocorrelation were identified for the posterior inferences.

## Discussion

In this study we aimed to explore the nature and extent of the association between potential risk factors and TB occurrence in wild ungulates and cattle in one of the most TB-prevalent regions of Spain referred to as Ciudad Real. The proportion of cattle farms becoming bTB positive in 2006, the mean number of hunting seasons in which the hunting estates of the municipality have been inspected and the apparent prevalence of TB in wild boar in 2006 were identified as the most relevant factors related to the TB occurrence in 2007 in Ciudad Real. These factors are useful predictors in planning disease control strategies. Results revealed that both livestock- and wildlife-related factors determine TB presence at spatial scale. Overall, the major risk factor for TB incidence in any given year is whether it was present or not, and at what level, in the previous year.

The probability of TB occurrence in Ciudad Real resembled the biogeography of the province as well as game and cattle farming distribution, with areas at high risk concentrated in the municipalities with a higher proportion of forest or natural habitats (Toledo Mountains in the Northwest corner, the Guadiana valley in the East, and Sierra Morena in the South), and the highest big game activity. In fact, the mean number of hunting seasons in which the hunting estates of the municipality have been inspected, which is an indicator of the hunting activity in the municipality, remained statistically significant in the model (see below). Usually, woodlands and savannah-like areas (locally called *dehesas*) are dedicated to big game, whereas marginal *dehesas* are located in the valleys in close proximity, where land is not appropriate for agriculture, are mainly dedicated to extensive livestock farming. This distribution, together with particular Mediterranean features that favour the wildlife-livestock interface (i.e. water areas or pasture sharing) may explain how TB could be transmitted from wildlife to livestock and vice versa (Figures 1 to 3). However, from this study we cannot infer the directionality of *M. bovis* transmission, i.e. whether wildlife reservoirs are infecting cattle, or whether cattle is the responsible of the circulation of *M. bovis* in both domestic and wild hosts. In fact, it could be the case that some municipalities with a high prevalence of TB in just one species (for example, wild boar) in 2007 may be purely correlated with a high prevalence of TB in only that species (in the example, with no red deer nor cattle infected in that municipality) the previous year. This would indicate no inter-species transmission in some regions. However, Ciudad Real municipalities with this scenario are an exception (9 out of 113) and many TB-infected regions presented TB cases in more than one species (44.19% in 2006 and 28.86% in 2007) (Table 2). This suggests a potential multi-species interaction scenario. Unfortunately, we cannot prove this because the final model did not include any interaction between variables related to hosts. Nevertheless, future studies should reveal whether the presence of the disease in wildlife is associated with increased presence of the disease in livestock and establish the directionality and extent of the TB inter-species transmission.

**Table 2:** Number of municipalities of Ciudad Real that were TB positive in 2006 and 2007 in either red deer (RD), wild boar (WB), cattle (Bov) or any of their combinations

	**RD**	**WB**	**Bov**	**RD + WB**	**RD + Bov**	**WB + Bov**	**RD + WB + Bov**	**Total**
TB positive in 2006	8	2	14	3	6	1	9	**43**
TB positive in 2007	3	1	16	3	4	0	1	**28**
Maintenance of the TB-positive status over the two years	1	1	7	2	4	0	1	**16**

The finding that municipalities with a high proportion of cattle farms becoming bTB positive in 2006 were at high risk for TB occurrence in 2007 (OR = 1.86; 95% CI = 1.06-3.39) is an indicator that TB until 2007 repeatedly tends to appear in the same municipalities. These findings are in agreement with the trend of incidence and persistence of bTB in South Central Spain during 2006–2009, particularly in Ciudad Real, observed in previous studies [14]. This indicates the persistence of the pathogen over consecutive sanitary campaigns despite the eradication efforts and the culling of positive animals. Herd-level research is needed to elucidate whether barriers imposed to prevent the transmission of *M. bovis* into bTB-free farms are not as efficient as expected, or diagnostics were not reliable enough in presumed free farms. Both of these risk factors have not been explored in this study. Most of the extensive farms and cattle census in Ciudad Real province are located in municipalities where some of the risk factors identified in this model may operate, such as wildlife TB reservoir abundance (see below). Although the TB inspection in Spain is compulsory in all cattle farms, these aspects also show that the efforts in controlling *M. bovis* infection in the whole cattle stock should not be relaxed, especially in extensive farms where enclosing and handling the whole stock is more difficult. Our final model suggests that the higher the number of cattle submitted to the sanitary campaigns, the higher the likelihood of the municipality to be TB positive the following year (OR = 1.94; CI = 1.11-3.40). As Brooks-Pollock and Keeling [20] reported, many elements associated with herd size could contribute to disease persistence within a herd, such as an increased number of movements or larger land coverage with increased risk of environmental contamination, or more densely stocked cattle. Nonetheless, this should be evaluated at the herd level.

Although the information regarding TB prevalence in wildlife is usually not available and more difficult to obtain than in cattle, we believe that it is also crucial to monitor the TB status of wild reservoirs in a region [19]. In fact, one of the significant covariates in the final model is the mean number of hunting seasons in which the hunting estates of the municipality have been inspected (OR = 1.69; 90% CI = 1.19-2.42), which is related to the intensity and sustainability of the hunting activity performed in each estate over time, and therefore the abundance of big game. In addition, the analysis revealed two additional major risk factors related to wild hosts: the apparent prevalence of TB in wild boar (OR = 1.94; 95% CI = 1.20-3.59) and the number of TB-positive red deer (OR = 1.69; 90% CI = 1.05-2.90) both in the previous year. The implication of wild boar and red deer in the TB presence and maintenance in South Central Spain has been previously suggested [10]; the wild boar showing higher TB prevalence than red deer [9,21]. In addition, wild boar has been proposed as the most relevant wild host of *M. bovis* in Spain [10] becoming a true TB reservoir in the Iberian Peninsula [22]. This importance is enhanced by its abundance in Europe, the high levels of infection observed in this species, its ability to disseminatethe pathogen, its scavenging behaviour and, mostly, its potential ability for crossing fences and contacting with livestock [22]. Given this situation, physical biosafety practices may serve as a means to cost-effectively prevent contact between wild and domestic mammals and thus TB inter-species transmission. This is particularly useful in those areas where the hunting industry plays an important role in the local economy, such as Ciudad Real. Also, any action conducted to reduce TB prevalence and mycobacteria spreading in wild ungulates should contribute to reduce the *M. bovis* transmission risk at the wildlife-livestock interface. Options include the management of host density, spatial aggregations at supplementary feeding sites or waterholes, the safe disposal of carrion and viscera by hunters, and oral vaccination [5,21].

There is evidence supporting the inter-species circulation of *M. bovis*[13] and several studies have recently confirmed the presence of the same *M. bovis* spoligotypes in cattle and wild fauna sharing the same area [8,11,22-26]. *M. bovis* transmission between wild and domestic animals may occur directly via close contact, or indirectly via contamination of food or the environment with *M. bovis*. Most of the bTB control programmes in developed countries are mainly directed towards livestock not including wildlife, even where it is obviously involved in disease maintenance. The lack of integral control strategies is more evident in extensive cattle, which is the sector mostly exposed to wildlife contacts [15]. This was supported previously [16] and in our results, since the proportion of cattle farms classified as extensive beef breeding farms (OR = 1.47; 90% CI = 1.02-2.14) and the number of farms devoted to bullfighting cattle were revealed as risk factors in the final model (although this latter covariate is not highly significant, 90% CI = 0.97-1.87, probably because there are scarce and very disperse observations). Both types of farms are integrated in natural pasture agrosystems in close proximity with wildlife. However, the inclusion of these two covariates related to extensive cattle farming reinforces the idea that sanitary programs should incorporate the wildlife interface, especially in those cattle farms most exposed to *M. bovis* infection, as has been done in Spain since 2011 ( http://rasve.mapa.es/Publica/Programas/Normativa.asp).

The Bayesian mixed effects multivariable logistic regression model was chosen as the best statistical approach to be used in this study. The reason is because the Bayesian methodology solves most of the problems faced by traditional statistical methods, e.g. the spatial autocorrelation, the potential dependence between the covariates or the incorporation of variables with few observations. The unit of analysis (i.e. municipality) was also considered adequate given the information that was available and the spatial level at which policies are taken. However, as any ecological study, some of the results may be biased as a consequence of the artificial grouping of observations and variables at the municipality level. In addition, differences in the municipality size may confound the interpretation of the maps, since the largest municipalities could appear at the highest risk only because they entail the majority of the observations [27]. Although variations in size among municipalities are not particularly large in the study area, we have used proportions in the majority of the covariates to minimize this problem. Despite these limitations, the approach used here is very useful for the exploration of spatially aggregated data and to highlight the most risky areas to perform more accurate analysis. The model proposed in this article is robust and consistent, as shown by the much lower DIC of the final model (72.32) and the small SD of S_i_ (0.1199) and U_i_ (0.0492). The robustness and consistence of this model allow to study the “TB hot spots” of the province in detail, since future epidemiological analyses at more local scale may be conducted.

## Conclusions

In this study we used a Bayesian mixed effects spatial model that controlled for unobserved spatial and non-spatial heterogeneity over-and-above a set of risk factor variables considering environment, wildlife and cattle epidemiological factors. Overall, the major risk factor for TB incidence at any given year is whether or not it was present, and at what level, in the previous year. Our results support that cattle and big game (namely wild boar and red deer) are both involved in the *M. bovis* circulation in this region, evidencing a clustered distribution in the western part and an endemicity of the disease in many municipalities. In addition, some characteristics of management and surveillance in this region seem to be linked to TB occurrence. All these findings may guide sanitary authorities to conduct efforts towards specific areas and epidemiological situations where the wildlife-livestock interface seems to critically hamper the definitive success of the bTB eradication program. We believe that the inclusion of wild animals in control and eradication programmes, and the development of efficient biosafety practices in extensive farms should be a priority to eradicate the disease in livestock. Further studies are needed in order to understand the complex multi-host interaction at the farm level to more precisely evidence directionality of transmission and risks factors associated to TB.

## Methods

### Study area and unit of analysis

**Figure 5:**
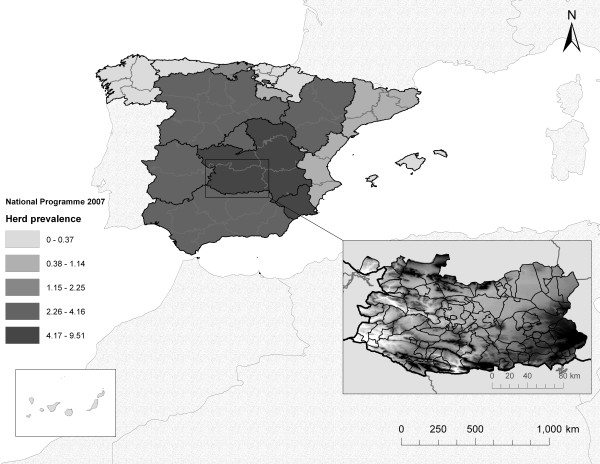
**Situation of bTB herd prevalences in Spain in 2007.** Autonomous Communities are marked in dark lines. In detail, the 102 municipalities of Ciudad Real (black lines), displayed in 113 polygons (because some municipalities are divided into several parts). The altitude of the province (ranging from 350 to 1261 m) is represented in a colour ramp (white to black); the highest areas are the Toledo Mountains (in the North), Sierra Morena (in the Southwest) and the southern part of the Central Plateau (in the East).

We performed the analysis in Ciudad Real province, South Central Spain (37° 13’ 48” N to 39° 31’ 43” N in latitude; 2° 25’ 54” W to 6° 34’ 06” W in longitude; 19,813 km^2^) (Figure 5). Ciudad Real has been traditionally one of the provinces with the highest levels of herd prevalence and incidence of bTB [14,15]. This province is flanked by two important mountainous zones, the Toledo Mountains and Sierra Morena, which are connected by the Guadiana river valley, a fragmented Mediterranean agriculture habitat. In this province both extensive livestock production and hunting industry are important. The smallest administrative level used for the bTB-decision making process, i.e. municipality, was used as the spatial unit of analysis (Figure 5). The province of Ciudad Real is divided into 102 municipalities, whose mean area is 175.23 ± SD 178.35 km^2^. For each municipality, we obtained data regarding TB sanitary status from 2002 to 2007, cattle and wild ungulate populations, management of farms/hunting estates and environmental factors (see below). 

### Data collection, case definition and exploratory cluster analysis

Information about bTB status in cattle was obtained from the official campaigns of 2002–2007 of the regional authorities. These campaigns, supported by the European Commission, are carried out in all the cattle farms of the province, including dairy, beef and bullfighting cattle. During these years, it was mandatory to perform the single IDTT, and all the reactors were culled [15]. In this context, all reactors to IDTT were considered “positive cases” in our study.

Data regarding wild ungulate (i.e. wild boar and red deer) abundance and TB presence were obtained from veterinary carcass inspection forms (from 1998–99 to 2007–08), which were also provided by the regional sanitary authorities. These forms register the number of animals shot per hunting event and the veterinary confiscation of carcasses due to macroscopic TB lesions at post-mortem examination [24]. Every carcass confiscated for this reason was considered to be a “positive case” of *M. bovis* infection in wild ungulates. From these reports, we also estimated an abundance population index for these wild species at municipality level and hunting pressure (see below).

We carried out an extensive literature review in order to capture and incorporate into the analysis the potential variables that may influence TB occurrence (i.e. risk or protective factors) (e.g. [2]). As a result, we gathered 50 variables (Table 3), which were grouped into three categories: (1) variables related to human demographics and environmental features; (2) variables related to wild ungulates; and (3) variables related to cattle herds. The first group of variables included the area of the municipalities, human-derived activity/presence (i.e. human population and density of roads) and water areas in each municipality. The second group included information concerning abundance of wild ungulates and the hunting pressure in each municipality. The relative abundances of wild boar and red deer at municipality level were calculated based on hunting yields (animals shot per municipality and hunting season). Hunting yields provide a coarse but feasible proxy of species abundance at broad spatial scales when absolute abundance values are lacking [28]. Within this group we also considered variables related to the inspection of TB in wild boar and red deer in the previous season (2006–07). The third category of variables included general information about the cattle sanitary plan developed in the municipality (i.e. number of cattle farms sampled, number of years in which the farms have been submitted to the plan, number of sampled animals) and variables related to the results of the bTB diagnostic test in cattle in the previous year (2006). This third category involved data about the animal species produced on farm (i.e. sheep, goats, pigs, or combinations of these three species with cattle), cattle movements per year (i.e. number of incoming movements, number of animals introduced on farm and average number of animals moved per shipment) and type of outdoor extensive production (i.e. breeding farms for meat production or farms for bullfighting cattle). In addition, this group contained data about the changes of the disease status of the farms in each municipality (i.e.becoming bTB positive/negative) from 2005 to 2006. 

**Table 3:** List of variables used to fit the model for TB occurrence in Ciudad Real

		**Polyserial correlation Rho**			**Phi coefficient**		
**Description of the variables**	**Quantitative standardized**	**Value**	***p*value**	**Dichotomous**	**Value**	***p*value**^**4**^	**Source**
1. Area of the municipality (km^2^)	Yes	0.343	<0.01	Yes	0.273	<0.01	A
2. Human population in the municipality in 2006 (persons)	Yes	−0.430	<0.01	**No**^**1**^	0.129	0.153	B
3. Male population in the municipality in 2006 (persons)	Yes	−0.427	<0.01	**No**^**1**^	0.129	0.153	B
4. Female population in the municipality in 2006 (persons)	Yes	−0.432	<0.01	**No**^**1**^	0.129	0.153	B
5. Area of water areas in the municipality (km^2^)	Yes	0.023	<0.01	Yes	0.173	0.074	C
6. Area of rivers in the municipality (km^2^)	Yes	0.456	<0.01	Yes	0.429	<0.01	C
7. Area of water areas and rivers in the municipality (km^2^)	Yes	0.263	<0.01	**No**^**2**^	0.429	<0.01	C
8. Area of roads in the municipality (km^2^)	Yes	0.256	<0.01	Yes	0.175	0.066	C
9. Proportion of water areas in the municipality (%)	Yes	0.010	<0.01	**No**^**1**^	0.111	0.248	*(C)
10. Proportion of rivers in the municipality (%)	**No**^**1**^	0.398	0.283	Yes	0.242	0.01	*(C)
11. Proportion of water areas and rivers in the municipality (%)	Yes	0.208	<0.01	**No**^**1**^	0.058	0.543	*(C)
12. Proportion of roads in the municipality (%)	**No**^**1**^	−0.078	0.235	**No**^**1**^	0.097	0.301	*(C)
13. Number of hunting estates in the municipality	Yes	0.615	<0.01	Yes	0.443	<0.01	D
14. Mean number of hunting seasons in which the hunting estates of the municipality have been inspected (max. 10 seasons)	Yes	0.593	<0.01	Yes	0.416	<0.01	D
15. Number of hunting events taking place in the municipality per hunting season 2006-07	Yes	0.512	<0.01	Yes	0.415	<0.01	D
16. Number of sampled red deer in the municipality in the hunting season 2006–07 – proxy of red deer relative abundance	Yes	0.408	<0.01	Yes	0.333	<0.01	D
17. Number of “TB-positive” red deer in the municipality in the hunting season 2006-07	Yes	0.588	<0.01	Yes	0.435	<0.01	D
18. Number of sampled wild boar in the municipality in the hunting season 2006–07 – proxy of wild boar relative abundance	Yes	0.344	<0.01	Yes	0.374	<0.01	D
19. Number of “TB-positive” wild boar in the municipality in the hunting season 2006-07	Yes	0.554	<0.01	Yes	0.406	<0.01	D
20. Apparent TB prevalence in red deer in the municipality in the hunting season 2006–07 (number of “TB-positive” animals/number of sampled animals)	Yes	0.559	<0.01	Yes	0.404	<0.01	*(D)
21. Apparent TB prevalence in wild boar in the municipality in the hunting season 2006–07 (number of “TB-positive” animals/number of sampled animals)	Yes	0.568	<0.01	Yes	0.406	<0.01	*(D)
22. Number of bovine farms in the municipality in 2006	Yes	0.667	<0.01	Yes	0.433	<0.01	D
23. Mean of the years in which the bovine farms of the municipality have been submitted to the sanitary plan (max. 6 years)	Yes	0.663	<0.01	Yes	0.441	<0.01	D
24. Number of sampled cattle in the cattle farms included in the sanitary plan in 2006	Yes	0.666	<0.01	Yes	0.569	<0.01	D
25. Number of bTB-positive cattle in the cattle farms included in the sanitary plan in 2006	Yes	0.501	<0.01	Yes	0.583	<0.01	D
26. Number of cattle farms with at least one bTB-positive animal in 2006	Yes	0.678	<0.01	**No**^**2**^	0.583	<0.01	D
27. Number of cattle farms with at least one bTB-positive animal relative to the total number of sampled farms in 2006 (%)	Yes	0.521	<0.01	**No**^**2**^	0.583	<0.01	*(D)
28. Apparent TB prevalence in the cattle farms of the municipality in 2006 (number of “TB-positive” animals/number of sampled animals)	Yes	0.237	<0.01	**No**^**2**^	0.583	<0.01	*(D)
29. Number of cattle farms becoming positive from 2005 to 2006	Yes	0.597	<0.01	Yes	0.504	<0.01	*(D)
30. Proportion of cattle farms becoming positive from 2005 to 2006 relative to the total number of sampled farms in both years (%)	Yes	0.523	<0.01	Yes	0.391	<0.01	*(D)
31. Number of cattle farms becoming negative from 2005 to 2006	Yes	0.414	<0.01	Yes	0.504	<0.01	*(D)
32. Proportion of cattle farms becoming negative from 2005 to 2006 relative to the total number of sampled farms in both years (%)	Yes	0.244	<0.01	Yes	0.391	<0.01	*(D)
33. Increment in the number of cattle farms becoming positive from 2005 to 2006 (number of farms becoming positive – number of farms becoming negative)	Yes	−0.081	<0.01	Yes	0.434	<0.01	*(D)
34. Number of farms devoted to bullfighting cattle in 2006	Yes	0.158	<0.01	**No**^**1**^	0.019	0.842	D
35. Proportion of farms devoted to bullfighting cattle in 2006 relative to the total number of sampled farms in 2006 (%)	Yes	−0.027	<0.01	**No**^**1**^	0.019	0.842	*(D)
36. Number of cattle farms classified as extensive beef breeding farms in 2006	Yes	0.656	<0.01	Yes	0.562	<0.01	D
37. Proportion of cattle farms classified as extensive beef breeding farms in 2006 relative to the total number of sampled farms in 2006 (%)	Yes	0.605	<0.01	Yes	0.466	<0.01	*(D)
38. Sum of animal entry movements in the cattle farms of the municipality in 2006	Yes	0.459	<0.01	Yes	0.425	<0.01	D
39. Number of animals moved in the animal entry movements in the cattle farms of the municipality in 2006	Yes	0.503	<0.01	**No**^**2**^	0.425	<0.01	D
40. Mean of animals moved in the animal entry movements in the cattle farms of the municipality in 2006	Yes	0.255	<0.01	**No**^**2**^	0.425	<0.01	*(D)
41. Number of cattle farms that also host goats in 2006	Yes	0.446	<0.01	Yes	0.344	<0.01	D
42. Number of cattle farms that also host sheep in 2006	Yes	0.620	<0.01	Yes	0.412	<0.01	D
43. Number of cattle farms that also host pigs in 2006	**No**^**3**^	0.508	<0.01	Yes	0.319	<0.01	D
44. Number of cattle farms that also host goats and pigs in 2006	**No**^**3**^	0.363	<0.01	Yes	0.234	0.017	D
45. Number of cattle farms that also host sheep and pigs in 2006	**No**^**3**^	0.454	<0.01	Yes	0.243	0.016	D
46. Proportion of cattle farms that also host goats in 2006 relative to the total number of sampled farms in 2006 (%)	Yes	0.265	<0.01	Yes	0.344	0.001	*(D)
47. Proportion of cattle farms that also host sheep in 2006 relative to the total number of sampled farms in 2006 (%)	Yes	0.236	<0.01	Yes	0.412	<0.01	*(D)
48. Proportion of cattle farms that also host pigs in 2006 relative to the total number of sampled farms in 2006 (%)	Yes	0.127	<0.01	Yes	0.319	<0.01	*(D)
49. Proportion of cattle farms that also host goats and pigs in 2006 relative to the total number of sampled farms in 2006 (%)	Yes	0.371	<0.01	Yes	0.234	0.017	*(D)
50. Proportion of cattle farms that also host sheep and pigs in 2006 relative to the total number of sampled farms in 2006 (%)	Yes	0.052	<0.01	Yes	0.243	0.016	*(D)

The variables were grouped into three categories: (1) variables for factors related to human demographics and environmental features (variables 1 to 12); (2). variables for factors related to wild animals (variables 13 to 21); and (3) variables for factors related to bovine herds (variables 22 to 50). Polyserial correlation’s Rho values, phi coefficients and *p*-values were estimated to each candidate variables.

1: excluded variables because of a *p*-value > 0.10.

2: excluded dichotomous variables because they have the same values as other related variables, due to the variable transformation process.

3: excluded variables because they have few observations different from 0.

4: *p*-value obtained from the chi-square test.

A: ArcGIS estimation.

B: National Institute of Statistics (January 1^st^ 2007).

C: Research Institute in Hunting Resources (IREC).

D: Castile-La Mancha region agriculture authorities.

*: Transformed from raw data.

The study presented here was designed as a longitudinal study in order to account for the inherent chronic character of the *M. bovis* infection and to provide policy makers with methods and results useful for the design of the “next year” eradication programmes. As a result, we tried to predict the TB status per municipality in one particular year by using information from that municipality in the previous year. The assumption here was that significant changes in factors such as wildlife population, cattle-wildlife contacts, or cattle movements will most likely influence the TB status in the following year rather than in the same year, due to the chronic nature of the disease (which implies lower effective transmission rates and lower positive skin responses to the IDTT). We selected the year 2007 because it was the most recent year for which complete information was available and because it represented one of the years with a meaningful increase in the incidence and prevalence of the disease in cattle and wildlife [15,16]. In addition, we explored spatial clusters of Ciudad Real municipalities at high risk by using the Bernoulli model [29]. In order to implement this model, each observation, i.e. municipality, was associated with a 1/0 variable denoting presence/absence of TB cases. The computation of this scan statistic was based on the application of circles of candidate clusters throughout the province. Sizes of the circles were varied up to a maximum size equal to the 50% of the population at risk. The expected probability of TB occurrence in Ciudad Real, under the null hypothesis of being homogenously distributed throughout the province, was computed using Monte Carlo simulation and significance (*p*-value < 0.05) of the candidate clusters was tested using a likelihood function [30].

### Data transformation and standardization

Before starting with the statistical analysis we standardized each variable to reduce correlation between predictors and to homogenize the results [31]. We also used the dichotomous transformed version of the quantitative predictors (converted using the median as a cut-off point, i.e. 1 if above the median, or 0 if below the median) to evaluate if this transformation improved the model fit. We used two correlation tests to select the best predictors for the response variable (i.e. TB occurrence in 2007). Considering that the response variable is dichotomous, the polyserial correlation was used for the quantitative variables (using the package “polycor” of the R language) [32]. Similarly, phi coefficients were calculated for the dichotomous variables (using the package “vcd” of the R language) [33]; significance of the phi values was evaluated using the chi-square test. Covariates correlated with the response variable in each test with a *p*-value > 0.10 were excluded from further analysis. In some cases different dichotomous variables had identical values, and thus the same relationship to the response variable. In this case we retained the least transformed or the most biologically significant ones, depending on the set of variables (see Table 3, exlusion criterion number 2). Thirdly, we excluded variables with few observations different from zero (i.e. less than 15%). As a result, a list of 81 potential risk factors or covariates was examined (Table 3).

### Bayesian model approach

A Bayesian mixed effects multivariable logistic regression model [34] was used to evaluate the association between the occurrence of *M. bovis* infection per municipality *i* and *k* epidemiological factors hypothesized to influence disease status in the region (*X*_i_):

(1) $$\log it\left(P_{i}\right)=\beta_{0}+\beta_{1}X_{1i}+\beta_{2}X_{2i}+\ldots \beta_{k}X_{\mathrm{ki}}+S_{i}+U_{i}$$

where, *p*_i_ denoted the posterior predicted probability of the municipality *i* to be TB positive, *X*_1i_ to *X*_ki_ indicated *k* candidate risk factors for the municipality *i* modelled as fixed effects with regression coefficients *β*_1_ to *β*_k_, respectively. Note that the response variable was whether or not *M. bovis* infection was detected in any animal (i.e. cattle, red deer or wild boar) in the municipality in 2007, which means that the *M. bovis* was present in that locality. Non-informative priors and hyper priors used were similar to those described in other works [31,35,36]. We used non-informative Normal priors defined by μ = 0 and σ^2^ = 4 for the intercept (*β*_0_) and the regression coefficients of the covariates (*β*_k_). The regression coefficients were set to show a lack of prior knowledge regarding the strength of any association between the covariates and the response variable. Spatially unstructured (U_i_) and structured (S_i_) random effects were included in the model to account for overdispersion and spatial autocorrelation [37]. We assumed for U_i_ a non-informative prior with mean = 0 and precision (τ_u_) ~ Gamma (0.5, 0.0005). In order to model S_i_, we used an intrinsic Gaussian autoregressive (CAR) structure, where the prior distribution of each S_i_ was dependent on the value of the response variable in every adjacent municipality [37] with a precision (τ_s_) ~ Gamma (0.5, 0.0005). This structure allows the final risk of each municipality to be smoothed, conditional on the risk of every municipality sharing borders with it [31]. Gamma distributions for both precisions (τ_u_ and τ_s_) were specified using a shape (r = 0.5) and a rate (μ = 0.0005).

We fitted the model using WinBUGS 1.4 [38] with 30,000 iterations, after burning out the first 500. The model was built using a purely forward selection routine, by trying all possible combinations introducing one covariate at each time, and subsequently introducing interactions between the covariates included. The deviance information criterion (DIC) value was used to select the best final model [38]. DIC is a method of generalized use for Bayesian model choice. This consists of the posterior mean deviance and a term referred to as the “effective number of parameters” (*pD*) that accounts for a penalty for over-parameterization of the model. Lower DIC values are preferred, indicating a better model, while a high *pD* value indicates excessive parameters in the model. When one variable was retained in the final model, all the variables related to it (both statatiscally and biologically, and in both continuous and categorical forms) were excluded from the analysis and not subsequently evaluated. Once the inclusion of an additional covariate increased the DIC value of the model, we stopped at the previous model, considered to be the final one. This final model was checked to confirm absence of autocorrelation in the predictions. Convergence of the model was also explored by using Gelman-Rubin plots [39]. All results obtained were mapped using ArcGIS 9.2.

## Abbreviations

bTB: Bovine Tuberculosis; IDTT: Intradermal Tuberculin Test; SD: Standard Deviation; S: Spatially Structured random effect; U: Spatially Unstructured random effect; DIC: Deviance Information Criteria; RR: Relative Risk; OR: Odds Ratio; CI: Credibility Interval.

## Authors’ contributions

VRP adapted the data, performed the model and drafted the manuscript. BML supervised the analysis and gave major advices about the methodology and interpretation of results. PA and JAB compiled the data on hunting bags and wildlife carcass inspection. BR and SRC compiled data and elaborated data on TB sanitary program in CLM, and together with PA also added value through introduction of critical technical considerations. JMSV, CG and JV were essential in the inter-institutional collaboration and contributed overall to the manuscript. All authors read, edit, review and approved the final manuscript.

## Author details

^1^VISAVET, Veterinary School, Complutense University of Madrid, Puerta de Hierro s/n, Madrid 28040, Spain. ^2^IREC Instituto de Investigación en Recursos Cinegéticos (CSIC-UCLM-JCCM), Ronda de Toledo s/n, Ciudad Real, 13071, Spain. ^3^Biogeography, Diversity and Conservation Research Team, University of Malaga, Málaga, 29071, Spain. ^4^Centre de Recerca en Sanitat Animal (CReSA), UAB-IRTA, Campus de la Universitat Autònoma de Barcelona, Bellaterra (Cerdanyola del Vallés), 08193, Spain.
